# Protective Effect of Alginate Microcapsules with Different Rheological Behavior on *Lactiplantibacillus plantarum* 299v

**DOI:** 10.3390/gels9090682

**Published:** 2023-08-24

**Authors:** Minerva Aurora Hernández-Gallegos, Javier Solorza-Feria, Maribel Cornejo-Mazón, José Rodolfo Velázquez-Martínez, María Eva Rodríguez-Huezo, Gustavo F. Gutiérrez-López, Humberto Hernández-Sánchez

**Affiliations:** 1 Departamento de Ingeniería Bioquímica, Escuela Nacional de Ciencias Biológicas, Instituto Politécnico Nacional, Av. Wilfrido Massieu esq. M. Stampa, UP Adolfo López Mateos, Ciudad de México CP 07738, Mexico; aurora.hernandez@ujat.mx (M.A.H.-G.); ggutierrezl@ipn.mx (G.F.G.-L.); 2 División Académica Multidisciplinaria de Jalpa de Méndez, Universidad Juárez Autónoma de Tabasco, Carretera Estatal Libre Villahermosa-Comalcalco Km 27 S/N, Ranchería, Jalpa de Méndez CP 86205, Mexico; 3 Centro de Desarrollo de Productos Bióticos del IPN, Km 8.5 carr. Yautepec-Jojutla, Yautepec CP 62731, Mexico; jsolorza@ipn.mx; 4 Departamento de Biofísica, Escuela Nacional de Ciencias Biológicas, Instituto Politécnico Nacional, Carpio y Plan de Ayala, Col. Santo Tomás, Ciudad de México CP 11340, Mexico; mcornejom@ipn.mx; 5 División Académica de Ciencias Agropecuarias, Universidad Juárez Autónoma de Tabasco, Carretera Villahermosa-Teapa Km. 25, Teapa CP 86291, Mexico; jose.velazquez@ujat.mx; 6 División Ingeniería Química y Bioquímica, Tecnológico de Estudios Superiores de Ecatepec, Ecatepec, Estado de México CP 55010, Mexico; eva_rodriguez_huezo@hotmail.com

**Keywords:** encapsulation, probiotics, alginate, thermal treatment, viscosity, *Lactiplantibacillus plantarum* 299v

## Abstract

Alginate encapsulation is a well-known technique used to protect microorganisms from adverse conditions. However, it is also known that the viscosity of the alginate is dependent on its composition and degree of polymerization and that thermal treatments, such as pasteurization and sterilization, can affect the structure of the polymer and decrease its protection efficiency. The goal of this study was to evaluate the protective effect of encapsulation, using alginates of different viscosities treated at different temperatures, on *Lactiplantibacillus plantarum* 299v under in vitro gastrointestinal conditions and cold storage at 4 °C and −15 °C, respectively. Steady- and dynamic-shear rheological tests were used to characterize the polymers. Thermal treatments profoundly affected the rheological characteristics of alginates with high and low viscosity. However, the solutions and gels of the low-viscosity alginate were more affected at a temperature of 117 °C. The capsules elaborated with high-viscosity alginate solution and pasteurized at 63 °C for 30 min provided better protection to the cells of *L. plantarum* 299v under simulated gastrointestinal and cold storage conditions.

## 1. Introduction

Probiotic bacteria provide many health benefits; however, in order to receive those benefits, the International Dairy Federation recommends a minimum number of 10^6^ CFU of microorganism per gram of food product or a daily intake of 10^8^ CFU [1,2,3,4]. Probiotics are defined as “live microorganisms that, when administered in adequate amounts, confer a health benefit on the host” [5]. Oftentimes, however, the number of viable probiotic bacteria that finally gets to the consumer is quite low due to numerous constraints encountered during the elaboration of food products, such as pH, cold storage post-acidification (in the case of fermented products such as yogurt), hydrogen peroxide production and storage temperature [6,7]. A second set of constraints is encountered during the gastrointestinal transit (pH, digestive enzymes, bile salts), which limits the survival of probiotics and their ability to provide health benefits [3,8,9]. Several methods have been used to enhance the viability of probiotics, including the selection of resistant strains, stress adaptation and incorporation of micronutrients and prebiotics, as well as microencapsulation [3]. Microencapsulation has been suggested by a large amount of research to be an efficient protective method for probiotic bacteria [10]. An ideal microcapsule would consist of a semi-permeable, thin, and strong membrane surrounding the probiotics, providing a particular and convenient micro-environment for the encapsulated microorganism while enhancing its viability [11,12,13]. The choice of materials is essential for an effective probiotic encapsulation strategy. Macromolecules such as polysaccharides (alginate, chitosan, gellan gum, xanthan gum, pullulan, and others), proteins and lipids have been used to encapsulate probiotics [2,12,14]. The most common encapsulation material is sodium alginate, due to its simplicity, non-toxicity, biocompatibility and low cost [1,11,13,15,16]. Currently, the industrial production of alginates totals more than 30,000 tons per year [17]. The use of alginate has additional advantages, such as the alleviation of intestinal inflammation and colitis in inflammatory bowel disease [18] and antimicrobial activity against pathogens [19]. Alginic acid, a polysaccharide extracted from algae, is a linear heteropolysaccharide formed by several units of D-mannuronic and L-guluronic acids, which can be crosslinked with multivalent cations such as Ca^2+^ or Ba^2+^ in a method known as ionic gelation. A mixture of microbial cells and sodium alginate solution in the presence of multivalent cations results in the formation of gel beads [11,13,20,21]. It is known that the gelation properties of alginates are dependent on experimental conditions, such as pH, temperature, crosslinker type and concentration, residence time and ionic strength [22]. The polymer solutions used in food, biotechnological and biomedical applications are usually sterilized to inactivate contaminant vegetative cells and spores [23], and this thermal treatment can induce depolymerization, oxidation or the formation of free radicals, which affects the integrity of the alginate molecules [24,25,26]. These treatments can also affect the alginate molecules’ properties, such as gel formation and its mechanical strength, swelling and stability behavior [23,27,28]. It is evident that the effect of high temperatures on alginates is well documented; however, to the best of our knowledge, the effect of these modifications on their probiotic encapsulation capacity has not been studied yet. The goal of this study was, then, to evaluate the protective effect of encapsulation, using alginates of different viscosities treated at different sterilizing temperatures, on *Lactiplantibacillus plantarum* 299v under in vitro gastrointestinal conditions and cold storage at 4 °C and −15 °C, respectively. The selection of this probiotic microorganism is based on their known benefits for gastrointestinal health, such as reduced flatulence and abdominal pain in patients with irritable bowel syndrome (IBS) and improved iron absorption in healthy individuals [29]

## 2. Results and Discussion

### 2.1. Rheological Characterization of the Aqueous Solutions of Sodium Alginate

**Figure 1:**
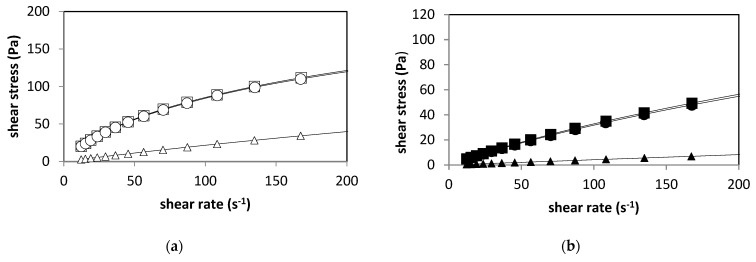
Flow curves of AD (**a**) and AF (**b**) sodium alginate after different thermal treatments showing non-Newtonian thinning behavior. AD sodium alginate treated at 117 °C/15 min (∆); 63 °C/30 min (○); untreated (□). AF sodium alginate treated at 117 °C/15 min (▲-closed symbols); 63 °C/30 min (●-closed symbols); untreated (■-closed symbols).

**Figure 2:**
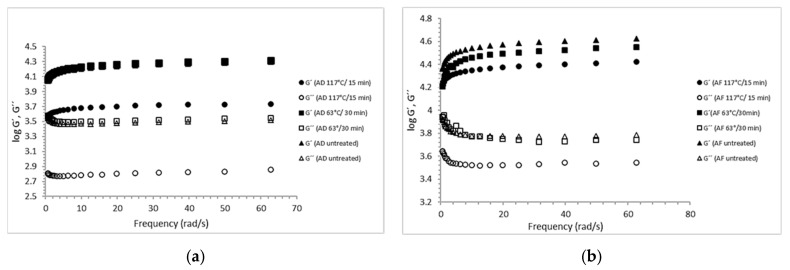
Dynamic frequency sweep for AD (**a**) alginate gels treated at 117 °C/15 min (○), 63 °C/30 min (□) and untreated gels (∆) and AF (**b**) alginate gels treated at 117 °C/15 min (○), 63 °C/30 min (□) and untreated gels (∆). Dynamic storage G′ and dynamic loss G″ moduli vs. frequency.

Figure 1 shows the classical flow curves, plotted as shear stress against shear rate. Shear-thinning flows are shown by curves and Newtonian flow by a straight line. The steady-shear flow tests used to determine the viscous properties of aqueous 2% sodium alginate solutions (AD and AF) indicated that they behaved as non-Newtonian fluids (Figure 1 and Figure 2) and that the flow curves data fitted well into the power-law model (R > 0.95) [30]:

τ = k γ^n^ (1)

where τ is the shear stress (Pa), k is the consistency coefficient (Pa·s^n^), γ is the shear rate (s^−1^) and n is the flow behavior index (dimensionless). If n = 1, the fluid is Newtonian; if n < 1, the fluid is shear-thinning (pseudoplastic) and if n > 1, the fluid is shear-thickening (swelling plastic or dilatant) [31].

The power-law model fitting parameters of sodium alginate suspensions with different thermal treatments are shown in Table 1.

It can be observed that *n* values of less than one were obtained for the untreated and pasteurized sodium alginate dispersions, confirming the findings of Ma et al. (2014), which stated that alginate suspensions exhibit non-Newtonian shear-thinning behavior [31]. The consistency coefficient could be defined as the shear stress at a shear rate of 1.0 s^−1^, and the greater its value, the thicker the suspension. It can be observed that the AD alginates (high-viscosity alginates) had the overall highest values for *k* for any given thermal treatment. The pasteurization treatment did not have a significant effect (*p* ≤ 0.05) on the rheological parameters; however, the sterilization treatment caused an important decrease in *k* and an increase in *n*. A value of *n* approaching 1 for both sterilized alginate dispersions was obtained, indicating that this high-temperature thermal treatment could cause extensive depolymerization such that the resulting suspension turned into an almost Newtonian fluid (see Figure 1). This effect was also observed by Yu et al. (2017). They observed the breaking of the main chains of alginate during the reaction of depolymerization by autoclaving, generating shorter chains that kept the same composition as the original chains [32]. Similary, Chansorioa et al. (2020) observed a significant reduction in the molecular weight (MW) and polydispersity index of 2% alginate solutions after autoclaving, and the reduction in MW is attributed to the high thermal fluxes during autoclave sterilization, which enable the breakdown of longer polymeric alginate chains [33].

In order to successfully encapsulate a microorganism, the selected wall material should exhibit non-Newtonian properties [30]; the data reported here indicate that the pasteurized aqueous solutions of alginate AD and AF satisfy this requirement. Newtonian liquids are unsuitable as encapsulating materials, as liquid droplets, instead of solid capsules, are formed during the process. On the other hand, sterilization is a necessary step that guarantees the destruction of the microorganisms naturally present in the alginates. However, this high-temperature process also leads to depolymerization of the molecule [24,28] and, as a consequence, a considerable decrease in viscosity, molecular weight and rheological properties. This, in turn, results in poor gelation, reduced mechanical properties and lowered water stability [23]. Pasteurization is a thermal treatment that, as shown in Figure 1 and Figure 2, does not affect the rheological properties of the alginate solutions; it is also very effective in reducing the microbial load of foods. In this case, pasteurization was able to reduce to zero the plate count of the alginate solutions , so it can be considered a valid method that guarantees the safety of foods with low initial microbial loads.

### 2.2. Dynamic Viscoelastic Properties of Calcium Alginate Gels

As a result of the applied shear stress amplitude sweep, in the linear viscoelastic region (LVR) of calcium alginate samples, the moduli (G′, G″) were independent of the stress applied. All the samples behaved as gel-like materials (G′ > G″), and LVR was found within the stress range from 0.5 to 5.0 Pa . Frequency sweep analysis of the gels used to determine G′ (storage or elastic modulus) and G″ (loss or viscous modulus) confirmed that all the calcium alginate gels behaved as predominantly solid-like materials (G′ > G″) over the frequency range applied, from 0.6283 to 62.83 rad/s [34,35]. Figure 2a shows the frequency profiles with non-linear (<10 rad/s) and linear (>10 rad/s) viscoelastic zones for AD alginate in which G′ > G″, but the value of the difference G′–G″ gets smaller as the frequency increases, as a result of an increase of G′; this is a typical behavior of viscoelastic gels [34]. The moduli values (G′, G″) of AF are higher than those of AD (Figure 2b), which suggests a higher strength of gels produced from AD. However, the frequency profiles of AF are overall similar to those of AD (G′ > G″). The sterilization treatment affects the storage and dynamic loss moduli to a higher extent for AD gels (Figure 2a) than for AF gels (Figure 2b).

**Figure 3:**
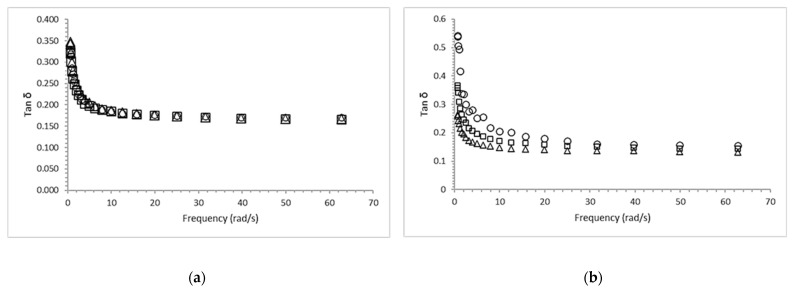
Viscoelastic loss tangent (tan δ) as a function of frequency indicated for alginate AD (**a**) treated at 117 °C/15 min (∆), 63 °C/30 min (○) and non-sterilized (□) and AF (**b**) treated at 117 °C/15 min (∆), 63 °C/30 min (○) and untreated gels (□).

The AD gels prepared from pasteurized and untreated sodium alginates had similar storage and dynamic loss moduli, whereas, in the case of AF, the gels prepared from the two thermally treated and the untreated samples had significantly different moduli (*p* ≤ 0.05). Alginates are susceptible to high temperatures, which may induce some degree of depolymerization [36] and a concomitant reduction in dynamic viscosity, as previously reported by Chansoria et al. (2020) [33]. Figure 3 shows that alginates are susceptible to thermal degradation at temperatures of 117 °C, since moist-heat treatment affects their functional properties, such as gelling ability [23,24]. Loss tangent (tan δ = G″/G′) is a measure of how much the stress and strain are out of phase with each other [35]. All frequency profiles are as expected, consistent with those of the loss tangent. A viscoelastic material may usually behave more elastic or solid (tan δ < 1) at higher frequencies and, in turn, more viscous or like a liquid (tan δ > 1) at lower frequencies [37]. As shown in Figure 3a,b, the loss tangent for alginates is <1 over the range of dynamic frequencies (overall range 0.133–0.539); that is why they behave as solids and amorphous-like polymers. However, the loss tangent profiles (Figure 3b) suggest that the temperature has some effect, especially on AF alginates [37,38,39].

### 2.3. Morphology of Empty and L. plantarum 299v-Loaded Alginate Capsules

**Figure 4:**
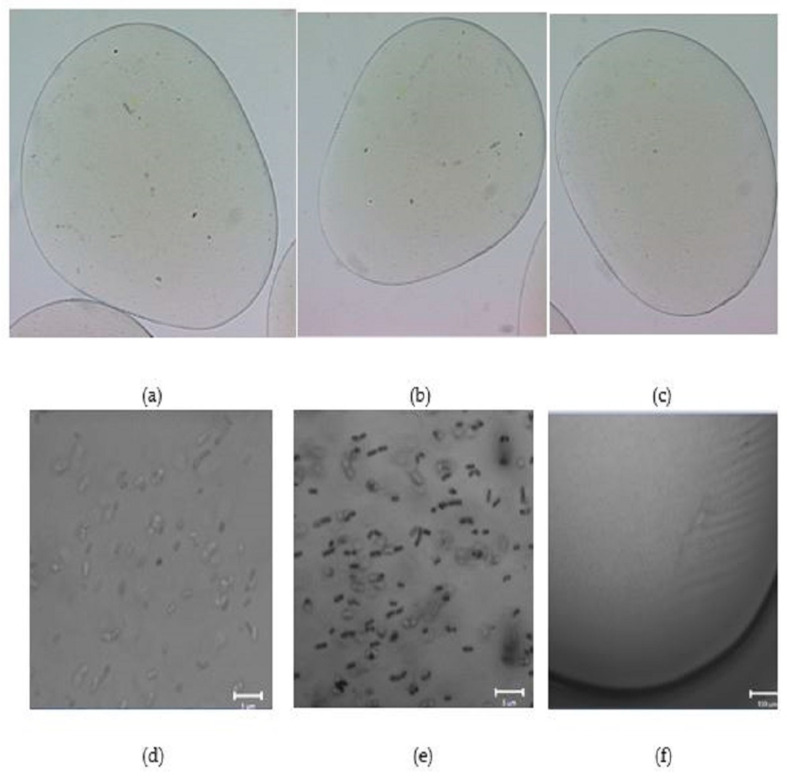
Morphology of capsules and cellular distribution in alginate AF. (**a**) show the morphology of a capsule of AF alginate treated at 117 °C/15 min, (**b**) AF alginate treated at 63 °C/15 min and (**c**) AF untreated. Images taken with a stereoscopic microscope. (**d**) shows the distribution of *Lactiplantibacillus plantarum* 299v in AF alginate capsule treated at 117 °C/15 min, (**e**) distribution *L. plantarum* 299v in AF alginate capsule treated 63 °C/15 min and (**f**) microorganism-free AF alginate capsule. Images taken with a confocal scanning microscope.

**Figure 5:**
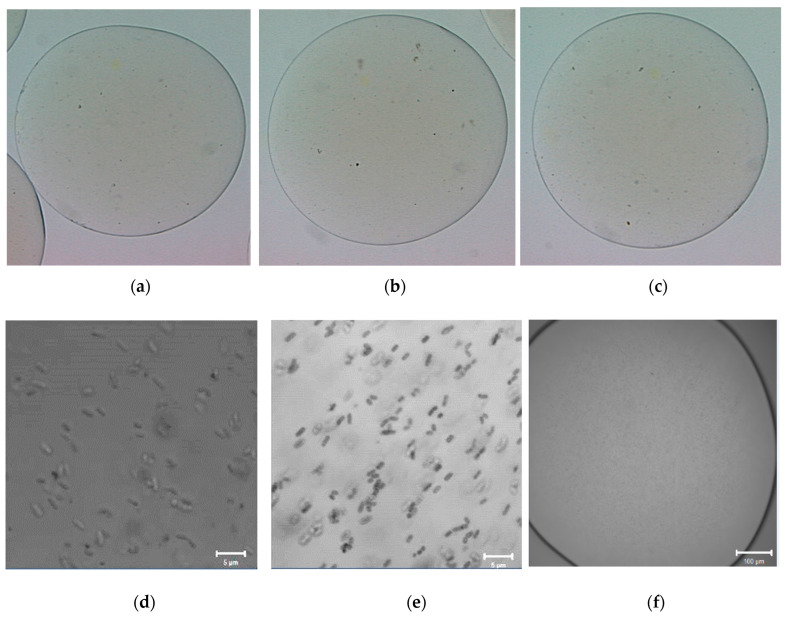
Morphology of capsules and cellular distribution in alginate AD. (**a**) show the morphology of a capsule of AD alginate treated at 117 °C/15 min, (**b**) AD alginate treated at 63 °C/15 min and (**c**) AF untreated. Images taken with a stereoscopic microscope. (**d**) shows the distribution of *Lactiplantibacillus plantarum* 299v in AD alginate capsule treated at 117 °C/15 min, (**e**) distribution *L. plantarum* 299v in AD alginate capsule treated 63 °C/15 min and (**f**) microorganism-free AD alginate capsule. Images taken with a confocal scanning microscope.

The AF and AD alginate capsules had an average diameter of 2250.85 µm. No significant differences between the diameters of microcapsules were found, (2.25 mm) (Figure 4a–c and Figure 5a–c). However, the microcapsules prepared with high-viscosity alginate (AD) solutions show better sphericity, while microcapsules prepared with low-viscosity alginate (AF) solutions show no spheric morphology. Furthermore, in both samples (AD and AF), the heat treatments affect the uniformity. The treatment at 63 °C is the one that has the least effect on the sphericity of the capsule. Figure 4d,e and Figure 5d,e show that the distribution of *Lactiplantibacillus plantarum* 299v is uniform for alginate AD and AF, with no differences in both treatments. Huang et al. 2023 reported similar diameters of alginate hydrogel beads. Furthermore, there is a relation between the sphericity, the uniform distribution and the protection within the hydrogel beads [40].

### 2.4. Effect of Thermal Treatment of the Alginate Solutions on Their Ability to Form Protective Capsules

**Figure 6:**
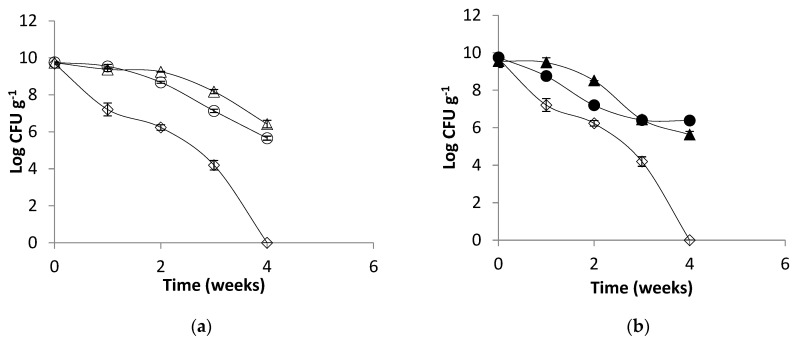
Viability of *Lactiplantibacillus plantarum* 299v under refrigerated storage (4 ± 1 °C) encapsulated in beads of alginate AD (**a**) treated at 117 °C/15 min (∆), 63 °C/30 min (○), free cells (◊) and AF (**b**) 117 °C/15 min (▲-closed symbols), 63 °C/30 min (●-closed symbols), free cells (◊).

Experiments were performed to evaluate the effect of thermal treatments of the aqueous solutions of alginate on their ability to improve the survival of *L. plantarum* 299v upon encapsulation. Alginate is a natural polymer that can easily be applied successfully for the microencapsulation of probiotic bacteria, protecting them from the low pH, bile salts and enzymes found during gastrointestinal transit [41]. It can also be added to fermented products and stored under refrigerated or freezing conditions [42]. Figure 6 shows the probiotic protection ability of calcium alginate beads after a 4-week storage period under refrigerated conditions at 4 °C. The free cells of *L. plantarum* 299v showed a complete loss of viability after 4 weeks of refrigerated storage. All the alginates provided good protection to the probiotic bacteria since counts of 10^6^–10^7^ CFU/g could be detected after 4months. Kong et al. (2005) concluded that a high viscosity of the sodium alginate solutions is not desirable in maintaining cell viability during the pre-gell/cell mixing process since the exposition of cells to high shear forces and mixing can lead to death [43]. However, Zhao et al. (2017) have reported that the survival of encapsulated *L. plantarum* ST-III was positively correlated with the mechanical strength of the microcapsules [44]. This highlights the importance of mechanical strength, which is dependent on the alginate’s rheological properties, in cell protection. In this study, the lower viscosity of the sterilized alginates did not have a greater effect on the probiotic survival after 4 weeks of refrigerated storage. The protection was as good as that provided by the capsules prepared with pasteurized alginates (*p* ≤ 0.05).

**Figure 7:**
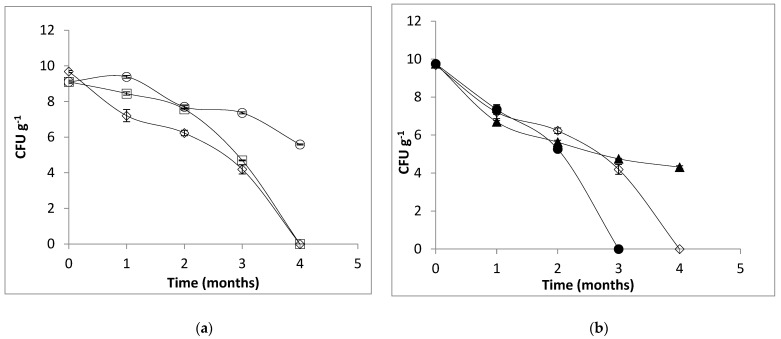
Viability of *L. plantarum* 299v under frozen storage (−15 ± 1 °C) encapsulated in beads of alginate AD (**a**) treated at 117 °C/15 min (∆), 63 °C/30 min (○), free cells (◊) and AF (**b**) treated at 117 °C/15 min (▲-closed symbols), 63 °C/30 min (●-closed symbols), free cells (◊).

In the case of the frozen storage at −15 °C experiment (see Figure 7), the cells inside the capsules prepared from AD alginate treated at 63 °C/30 min showed the highest rate of survival after 4 months (10^6^ CFU/g), followed by the cells in the capsules prepared with AF alginate treated at 117 °C/15 min (10^5^ CFU/g). The free cells and the other encapsulated cells showed a total loss of viability after the 4 months of frozen storage. Loss of viability during frozen storage of encapsulated probiotic cells was also reported by Tsen et al. (2007) in the case of *Lacticaseibacillus rhamnosus* [45]. Lower storage temperatures are associated with extended viability of the preserved samples when compared with the unencapsulated cells. However, when temperatures are in the range of −15 °C to 4 °C, the cells can be considered metabolically active, and the storage conditions could lead to cell death or loss of viability since the diffusion of nutrients to the cell is limited by temperature and ice. In addition, the existence of ice or unfrozen water will lead to swelling of the microcapsules, which may alter their surface morphology and cause irreversible rupturing of the walls [46,47].

### 2.5. Effect of Temperature on Alginate Beads and Survival of Microencapsulated Cells in Simulated Gastric Juice

**Figure 8:**
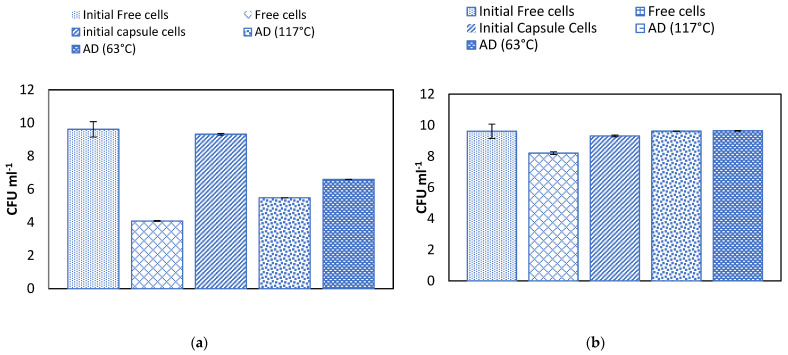

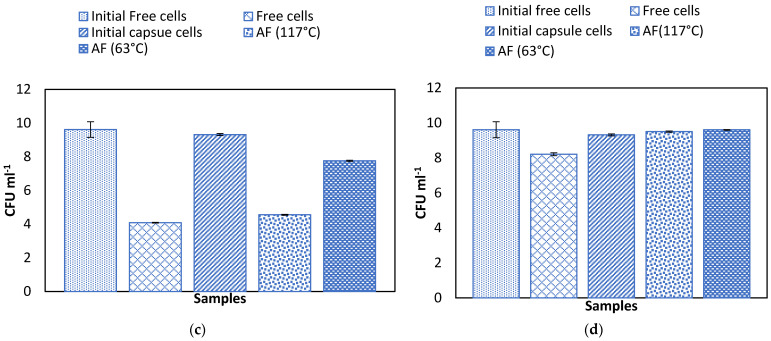
Effect of alginate AD (117 and 63 °C), AF (117 and 63 °C) and free cells on stability of encapsulated *L. plantarum* 299v in simulated gastrointestinal fluids. (**a**) Effect of gastric fluid on microcapsules of AD alginate. (**b**) Effect of intestinal fluid on microcapsules of AD alginate. (**c**) Effect of gastric fluid on microcapsules of AF alginate. (**d**) Effect of intestinal fluid on microcapsules on alginate AF.

The bacteria encapsulated in the pasteurized AD and AF alginates (63 °C, 30 min) survived well under the simulated gastric conditions compared to the cells encapsulated in sterilized AD and AF alginates (117 °C, 15 min) and non-encapsulated cells (See Figure 8a,c), reaching final concentrations between 10^7^ and 10^8^ CFU/g. In the case of the intestinal conditions, all the alginates adequately protected the probiotic cells, with a final survival rate of 100% (See Figure 8b,d). There are reports indicating that capsules of alginate effectively protect probiotic microorganisms [41,48] under gastrointestinal conditions. Leo et al. (1990) observed that sterilization modified the gel structure with consequences in mass transfer, cell growth and product formation [24]. Cook et al. (2012) described the mechanism of the protection that the alginate capsules provide to the cells in terms of a buffering effect in which the activity of the protons is reduced by the dense polymer environment [44,49]. Zhao et al. (2017) studied the swelling/shrinking behavior and permeability of alginate microcapsules on the survival of *L. plantarum* ST-III and concluded that the microbeads with higher mechanical strength could better tolerate the osmotic pressure, avoiding the diffusion of digestive fluids into the microcapsules and thus decreasing damage to the cells [44]. High-temperature moist-heat treatments deeply affect the gelling ability of alginate solutions and also decrease the mechanical strength of the formed gels, thus diminishing their cell-protective capacity [41].

## 3. Conclusions

The present study showed that the viscosity of thermally treated sodium alginate solutions had an important influence on the rheological properties. The involved solutions behaved overall as non-Newtonian samples. Additionally, all alginate gels behaved as weak viscoelastic rheological systems with predomination of their elastic component. The microcapsules prepared from pasteurized high-viscosity alginate (AD) solutions enhanced the survival of the probiotic bacteria *L. plantarum* 299v under refrigerated and frozen storage and gastrointestinal conditions, thereby maximizing the health benefits. It is then corroborated that autoclave sterilization of alginate, which leads to almost Newtonian behavior, decreases the protective effects toward microorganisms. According to these results, pasteurization or filtration can be recommended to sterilize sodium alginate solutions to maximize the survival of encapsulated probiotics.

## 4. Materials and Methods

### 4.1. Materials

De Man Rogosa Sharpe (MRS) lactobacilli broth agar was purchased from BD DIFCO (Detroit, MI, USA). High-viscosity sodium alginate (AD) with 60.5% guluronic acid content was purchased from Danisco Mexico, S.A. of C.V. (Grindsted alginate FD175). Low-viscosity sodium alginate (AF) from brown algae (Fluka), calcium chloride (CaCl_2_) and sodium chloride (NaCl) were purchased from Sigma-Aldrich (St. Louis, MO, USA).

### 4.2. Rheology of Aqueous Solutions of Sodium Alginate

For rheological measurements, the aqueous systems of sodium alginate (AD and AF) were prepared as 2 % *w/v* dispersions in demineralized water at room temperature and either pasteurized at 63 °C for 30 min or sterilized at 117 °C for 15 min. A rheometer AR1000-N (TA Instruments Co., New Castle, DE, USA) was used to determine the flow curves. It was used with a cone-plate measuring system of 60 mm diameter, with an angle between the surface of the cone and the plate of 1°, in controlled shear rate mode, at shear rates of 10–500 s^−1^ and a constant temperature of 20 °C [30,50,51].

### 4.3. Dynamic Shear Rheology of Calcium Alginate Gels

Gels were prepared from 2% *w/v* aqueous solutions of alginates (AD and AF), as described above. The solutions were packed in a cellulose dialysis membrane and submerged in 0.2 M CaCl_2_ for 48 h. The gels were cut in slices of around 1.5 mm thickness with a microtome (Bresser, Germany). The gel slices were stored in saline (NaCl) solution 0.9% *w/v* [52]. The dynamic rheological properties were measured at 25 °C using a rheometer AR1000-N (TA Instruments Co., DE, USA) with normal force and a parallel plate system (20 mm diameter). Dynamic shear data were obtained as follow: shear stress amplitude sweep was run in the range of 0.1 to 20 Pa, with a frequency of 6.283 rad/s, to find the linear viscoelastic region (LVR). Frequency sweeps were run over the range of 0.6283 to 62.83 rad/s, with a shear stress value of 2 Pa, which was within the LVR. The dynamic storage modulus (G′) and dynamic loss modulus (G″) (Pa), as well as the dynamic mechanical loss tangent (tan δ) defined by G″/G′, were obtained in order to be able to characterize the elastic and viscous responses of the gels through the dynamic frequency sweeps [26,34,51]

### 4.4. Bacterial, Growth Conditions and Preparation of Cell Suspensions

A pure culture of the probiotic bacteria *Lactiplantibacillus plantarum* 299v was isolated from the commercial product Protransitus LP (Salvat, Barcelona, Spain). The bacteria were cultured at 37 °C for 12 h under aerobic conditions in MRS broth. The fresh culture was prepared by adding 2% *w/v* inoculum to the MRS broth and incubating at 37 °C for 12 h. The cell concentrate (CC) was harvested by centrifugation (3000× *g* for 15 min) and suspended in sterile saline solution (0.9% *w*/*v*) at a concentration of about 1 × 10^9^ CFU/mL and centrifuged again under the same condition. Washed cells were suspended in 10% of the initial volume and stored at 4 °C until used. Longer-term stocks were preserved in 20% *v/v* glycerol at −70 °C. Fresh cell suspensions were prepared for each experiment and plate counted in MRS agar. Plates were incubated at 37 °C for 24 h [10,52,53].

### 4.5. Microencapsulation

Ionic gelation was used for the microencapsulation process. The aqueous solutions (2% *w*/*v*) of sodium alginate were sterilized (117 °C for 15 min) or pasteurized (63 °C for 30 min) to study their encapsulation capacity [53,54]. The CC, prepared as described above, was added (10% *v*/*v*) to the sodium alginate solution. The mixture of sodium alginate and CC was dripped through a peristaltic pump with a 0.5 mm diameter tubing discharger and a flow of 3 mL/min into a 0.2 M CaCl_2_ solution with continuous stirring (100 rpm). The beads were allowed to rest in the 0.2 M CaCl_2_ solution for 20 min for hardening purposes [54]. Thereafter, the beads were washed and suspended in saline solution (0.9% *w*/*v*) until used.

### 4.6. Morphology of Empty and L. plantarum 299v-Loaded Alginate Capsules

The morphology of capsules was evaluated using a stereoscopic microscope (Nikon, SMZ1500); the image was captured using the software MetaMorph version 6.1 (1992–2003 Universal Imaging Corp. Bedford Hills, NY, USA). The size of the cells was measured using the Imagen J software version 1.39 (National Institutes of Health, Bethesda, MD, USA). The pixel size of the image was 6.58 Micras per pixel.

In the case of the loaded capsules, the sample was analyzed at a 405 nm excitation wavelength laser and 2% transmittance, using a confocal scanning microscope, LSM 710 NLO (Carl Zeiss, Jena, Germany). The image was taken with global objects, Plan-Apochromat 20×/0.8 M27, and point objects, Plan-Apochromat 40×/1.3 Oil DIC M27. Image analyses were carried out with the software ZEN 2010 (Carl Zeiss, Alemania, Germany).

### 4.7. Protective Ability of the Alginate Capsules

The survival rate of the probiotic bacteria *L. plantarum* 299v was evaluated under different non-sequential stressful conditions, as described below.

Refrigerated storage: The beads were suspended in sterile saline solution (9 g/L) and stored at 4 °C for 4 weeks. The survival of the cells was determined by plate counting in MRS-cysteine agar after dissolving the capsules in 0.1 M sodium citrate. The survival was compared to that of free cells under the same conditions.

Frozen storage: The experiment was similar to that of refrigerated storage, but, in this case, the beads were stored a −15 °C for 4 months.

Simulated gastrointestinal tract conditions: The gastric solution (GS) was prepared by dissolving pepsin (0.3 g/L) and NaCl (2 g/L) in phosphate buffer (0.05 M) adjusted at pH 2.3 with concentrated HCl. The capsules or free cells were suspended in this solution and incubated at 37 °C for 1 h with orbital shaking (150 rpm). The intestinal solution (IS) was prepared by dissolving pancreatin (1 g/L) and bile salts (4.5 g/L) in phosphate buffer (0.05 M) adjusted to pH 7.4 with 0.1 M NaOH. The capsules or free cells were suspended in this solution and incubated at 37 °C for 3 h with orbital shaking (150 rpm).

### 4.8. Statistical Analysis

Data are reported as the mean ± standard deviation (SD) (n ≥ 3) and were statistically analyzed by one-way ANOVA (analysis of variance) using MS-Excel 365 software (2008 Microsoft, Redmond, WA, USA). A value of *p* ≤ 0.05 was considered to be significant. If significance existed, a post hoc analysis was performed using the Tukey test to evaluate significance between individual data sets.

## Figures and Tables

**Table 1:** Power-law fitting parameters of sodium alginate solutions after different thermal treatments.

Sodium Alginate	Power-Law Model Fitting Parameters		
	n	k	R
AD untreated	0.514	7.627	0.998
AD pasteurized	0.513	7.551	0.998
AD sterilized	0.849	0.434	0.999
AF untreated	0.754	1.001	0.999
AF pasteurized	0.756	0.961	0.999
AF sterilized	0.986	0.044	1.000

## Data Availability

Data available on request from the authors.

## References

[1] Chávarri M., Marañón I., Ares R., Ibáñez F.C., Marzo F., Villarán M.C. (2010). Microencapsulation of a probiotic and prebiotic in alginate-chitosan capsules improves survival in simulated gastro-intestinal conditions. Int. J. Food Microbiol..

[2] Dong Q.Y., Chen M.Y., Xin Y., Qin X.Y., Cheng Z., Shi L.E., Tang Z.X. (2013). Alginate-based and protein-based materials for probiotics encapsulation: A review. Int. J. Food Sci. Technol..

[3] Rokka S., Rantamäki P. (2010). Protecting probiotic bacteria by microencapsulation: Challenges for industrial applications. Eur. Food Res. Technol..

[4] Rodriguez-Huezo M.E., Lobato-Calleros C., Reyes-Ocampo J.G., Sandoval-Castilla O., Perez-Alonso C., Pimentel-Gonzales D.J. (2011). Survivability of entrapped Lactobacillus rhamnosus in liquid-and gel-core alginate beads during storge and simulated gastrointestinal conditions. Rev. Mex. Ing. Quim..

[5] Hill C., Guarner F., Reid G., Gibson G.R., Merenstein D.J., Pot B., Salminen S. (2014). Expert consensus document: The International Scientific Association for Probiotics and Prebiotics consensus statement on the scope and appropriate use of the term probiotic. Nat. Rev. Gastroenterol. Hepatol..

[6] Martin M.J., Lara-Villoslada F., Ruiz M.A., Morales M.E. (2013). Effect of unmodified starch on viability of alginate-encapsulated Lactobacillus fermentum CECT5716. LWT Food Sci. Technol..

[7] Randazzo C.L., Pitino I., Licciardello F., Muratore G., Caggia C. (2013). Survival of Lactobacillus rhamnosus probiotic strains in peach jam during storage at different temperatures. Food Sci. Technol..

[8] Doherty S.B., Gee V.L., Ross R.P., Stanton C., Fitzgerald G.F., Brodkorb A. (2011). Development and characterisation of whey protein micro-beads as potential matrices for probiotic protection. Food Hydrocoll..

[9] Fritzen-Freire C.B., Prudêncio E.S., Pinto S.S., Muñoz I.B., Amboni R.D.M.C. (2013). Effect of microencapsulation on survival of Bifidobacterium BB-12 exposed to simulated gastrointestinal conditions and heat treatments. LWT-Food Sci. Technol..

[10] Nag A., Das S. (2013). Effect of trehalose and lactose as cryoprotectant during freeze-drying, in vitro gastro-intestinal transit and survival of microencapsulated freeze-dried Lactobacillus casei 431 cells. Int. J. Dairy Technol..

[11] Kailasapathy K. (2002). Microencapsulation of probiotic bacteria: Technology and potential applications. Curr. Issues Intest. Microbiol..

[12] Okuro P.K., Thomazini M., Balieiro J.C.C., Liberal R.D.C.O., Fávaro-Trindade C.S. (2013). Co-encapsulation of *Lactobacillus acidophilus* with inulin or polydextrose in solid lipid microparticles provides protection and improves stability. Food Res. Int..

[13] Shi L.E., Li Z.H., Li D.T., Xu M., Chen H.Y., Zhang Z.L., Tang Z.X. (2013). Encapsulation of probiotic Lactobacillus bulgaricus in alginate–milk microspheres and evaluation of the survival in simulated gastrointestinal conditions. J. Food Eng..

[14] Hashmi S.A.S., Deshpande H.W., Farooqui A.S., Syed H.M., Sontakke M.D. (2018). Microencapsulation of Probiotic Culture Beads by Using Modified Psyllium Husk. Int. J. Curr. Microbiol. App. Sci..

[15] Chandramouli V., Kailasapathy K., Peiris P., Jones M. (2004). An improved method of microencapsulation and its evaluation to protect *Lactobacillus* spp. in simulated gastric conditions. J. Microbiol. Methods.

[16] Gonzalez-Pujana A., Orive G., Pedraz J.L., Santos-Vizcaino E., Hernandez R.M., Rehm B., Moradali M. (2018). Chapater 3-Alginate Microcapsules for Drug Delivery. Alginates and Their Biomedical Applications.

[17] Rosiak P., Latanska I., Paul P., Sujka W., Kolesinska B. (2021). Modification of Alginates to Modulate Their Physic-Chemical Properties and Obtain Biomaterials with Different Functional Properties. Molecules.

[18] Pi Y., Zhang X., Wu Y., Wang Z., Bai Y., Liu X., Han D., Zhao J. (2022). Alginate Alleviates Dextran Sulfate Sodium-Induced Colitis by Promoting Bifidobacterium animalis and Intestinal Hyodeoxycholic Acid Synthesis in Mice. Microbiol. Spectr..

[19] Hurtado A., Aljabali A.A.A., Mishra V., Tambuwala M.M., Serrano-Aroca Á. (2022). Alginate: Enhancement Strategies for Advanced Applications. Int. J. Mol. Sci..

[20] Klinkenberg G., Lystad K.Q., Levine D.W., Dyrset N. (2001). pH-controlled cell release and biomass distribution of alginate-immobilized *Lactococcus lactis* subsp. lactis. J. Appl. Microbiol..

[21] Kawaguti H.Y., Sato H.H. (2011). Production of isomaltulose obtained by *Erwinia* sp. cells submitted to different treatments and immobilized in calcium alginate. Food Sci. Technol..

[22] Bennacef C., Desobry-Banon S., Probst L., Desobry S. (2021). Advances on alginate use for spherification to encapsulate biomolecules. Food Hydrocoll..

[23] Munarin F., Bozzini S., Visai L., Tanzi M.C., Petrini P. (2013). Sterilization treatments on polysaccharides: Effects and side effects on pectin. Food Hydrocoll..

[24] Leo W.J., McLoughlin A.J., Malone D.M. (1990). Effects of sterilization treatments on some properties of alginate solutions and gels. Biotechnol. Prog..

[25] Serp D., Mueller M., Von Stockar U., Marison I.W. (2002). Low-temperature electron microscopy for the study of polysaccharide ultrastructures in hydrogels. II. Effect of temperature on the structure of Ca^2+^-alginate beads. Biotechnol. Bioeng..

[26] Funami T., Fang Y., Noda S., Ishihara S., Nakauma M., Draget K.I., Nishinari K., Phillips G.O. (2009). Rheological properties of sodium alginate in an aqueous system during gelation in relation to supermolecular structures and Ca^2+^ binding. Food Hydrocoll..

[27] Mano J.F. (2003). Propiedades térmicas de los polímeros en la enseñanza de la ciencia de materiales e ingeniería. Estudios DSC sobre Poli (Tereftalato de Etileno). J. Mater. Educ..

[28] Hu T., Yang Y., Tan L., Yin T., Wang Y., Wang G. (2014). Effects of gamma irradiation and moist heat for sterilization on sodium alginate. Biomed. Mater. Eng..

[29] Nordström E.A., Teixeira C., Montelius C., Jeppsson B., Larsson N. (2021). *Lactiplantibacillus plantarum* 299v (LP299V^®^): Three decades of research. Benef. Microbes.

[30] McMaster L.D., Kokott S.A. (2005). Micro-encapsulation of *Bifidobacterium lactis* for incorporation into soft foods. World J. Microbiol. Biotechnol..

[31] Ma J., Lin Y., Chen X., Zhao B., Zhang J. (2014). Flow behavior, thixotropy and dynamical viscoelasticity of sodium alginate aqueous solutions. Food Hydrocoll..

[32] Yu H., Cauchois G., Schmitt J.-F., Louvet N., Six J.-L., Chen Y., Rahouadj R., Huselstein C. (2017). Is there a cause-and-effect relationship between physicochemical properties and cell behavior of alginate-based hydrogel obtained after sterilization. J. Mech. Behav. Biomed. Mater..

[33] Chansoria P., Narayanan L.K., Wood M., Alvarado C., Lin A., Shirwaiker R.A. (2020). Effects of autoclaving, etoh, and uv sterilization on the chemical, mechanical, printability, and biocompatibility characteristics of alginate. ACS Biomater. Sci. Eng..

[34] Moresi M., Bruno M., Parente E. (2004). Viscoelastic properties of microbial alginate gels by oscillatory dynamic tests. J. Food Eng..

[35] Saha D., Bhattacharya S. (2010). Hydrocolloids as thickening and gelling agents in food: A critical review. Food Sci. Technol..

[36] Holme H.K., Davidsen L., Kristiansen A., Smidsrød O. (2008). Kinetics and mechanisms of depolymerization of alginate and chitosan in aqueous solution. Carbohydr. Polym..

[37] Dartois A., Singh J., Kaur L., Singh H. (2010). Influence of Guar Gum on the In Vitro Starch Digestibility—Rheological and Microstructural Characteristics. Food Biophys..

[38] Gargallo L., Radic’ D. (1980). Chapter 2: Viscoelastic Behaviour of Polymers. Physicochemical Behavior and Supramolecular Organization of Polymers.

[39] Fuongfuchat A., Jamieson A.M., Blackwell J., Gerken T.A. (1996). Rheological studies of the interaction of mucins with alginate and polyacrylate. Carbohydr. Res..

[40] Huang Y., Zhang L., Hu J., Liu H. (2023). Improved Loading Capacity and Viability of Probiotics Encapsulated in Alginate Hydrogel Beads by In Situ Cultivation Method. Foods.

[41] Shori A.B. (2017). Microencapsulation Improved Probiotics Survival during Gastric Transit. HAYATI J. Biosci..

[42] Brinques G.B., Ayub M.A.Z. (2011). Effect of microencapsulation on survival of Lactobacillus plantarum in simulated gastrointestinal conditions, refrigeration, and yogurt. J. Food Eng..

[43] Kong H.J., Smith M.K., Mooney D.J. (2003). Designing alginate hydrogels to maintain viability of immobilized cells. Biomaterials.

[44] Zhao M., Qu F., Wu Z., Nishinari K., Phillips G.O., Fang Y. (2017). Protection mechanism of alginate microcapsules with different mechanical strength for *Lactobacillus plantarum* ST-III. Food Hydrocoll..

[45] Tsen J.-H., Huang H.-Y., King V.A.-E. (2007). Enhancement of freezing-resistance of Lactobacillus rhamnosus by the application of cell immobilization. J. Gen. Appl. Microbiol..

[46] Liu Y.G., Bai Y., Wang S.B., Chen A.Z., Wu W.G. (2013). Effect of the Storage Temperatures on BRL-encapsulated-alginate-PLO-alginate (APornA) Microcapsules. J. Biomim. Biomater. Tissue Eng..

[47] Rajam R., Kumar S.B., Prabhasankar P., Anandharamakrishnan C. (2015). Microencapsulation of Lactobacillus plantarum MTCC 5422 in fructooligosaccharide and whey protein wall systems and its impact on noodle quality. J. Food Sci. Technol..

[48] Lee K.-Y., Heo T.-R. (2000). Survival of Bifidobacterium longum immobilized in calcium alginate beads in simulated gastric juices and bile salt solution. Appl. Environ. Microbiol..

[49] Cook M.T., Tzortzis G., Charalampopoulos D., Khutoryanskiy V.V. (2012). Microencapsulation of probiotics for gastrointestinal delivery. J. Control. Release.

[50] Rezende R.A., Bártolo P.J., Mendes A., Filho R.M. (2009). Rheological behavior of alginate solutions for biomanufacturing. J. Appl. Polym..

[51] Andriamanantoanina H., Rinaudo M. (2010). Characterization of the alginates from five madagascan brown algae. Carbohydr. Polym..

[52] Annan N.T., Borza A.D., Hansen L.T. (2008). Encapsulation in alginate-coated gelatin microspheres improves survival of the probiotic Bifidobacterium adolescentis 15703T during exposure to simulated gastro-intestinal conditions. Food Res. Int..

[53] González-Sánchez F., Azaola A., Gutiérrez-López G.F., Hernández-Sánchez H. (2010). Viability of microencapsulated Bifidobacterium animalis ssp. lactis BB12 in kefir during refrigerated storage. Int. J. Dairy Technol..

[54] Gbassi G.K., Vandamme T., Ennahar S., Marchioni E. (2009). Microencapsulation of Lactobacillus plantarum spp in an alginate matrix coated with whey proteins. Int. J. Food Microbiol..

